# A quarter-century of HIV prevention intervention efforts among children and adolescents across the globe

**DOI:** 10.1080/21642850.2014.889572

**Published:** 2014-02-27

**Authors:** Bonita Stanton, Xiaoming Li

**Affiliations:** ^a^ School of Medicine, Wayne State University, Suite 1261 Scott Hall, Canfield Street, Detroit, MI48201, USA; ^b^ Pediatric Prevention Research Center, Wayne State University School of Medicine, 4707 St. Antoine, Suite W534, Detroit, MI48201-2196, USA

**Keywords:** HIV/AIDS, implementation science, children and adolescents

## Abstract

In 1988 a group of pediatricians, developmental, clinical, child and social psychologists, anthropologists and health educators began researching in Baltimore, Maryland, on an Human Immunodefiency Virus (HIV) prevention intervention, Focus on Youth (FOY). Over the next 25 years, the questions being addressed by FOY reflected those of the global HIV research experience. During the first phase, the questions being addressed by the broader research community included: *Can HIV risk behaviors be purposefully impacted by behavioral interventions? If so, how do successful interventions differ from those that are not effective? Are theory-based interventions more likely to be effective than information-only-based interventions? Can theories be translated into culturally and developmentally appropriate interventions including those that are appropriate for children and adolescents? Should parents be involved – and if so, how?* During its next phase, the FOY team increasingly became concerned with a disturbing reality. A large number of interventions had been developed and some had been shown to have evidence of impact. But virtually all of these interventions had been conducted in the USA or Europe. The questions facing researchers included: *With the global burden of HIV disproportionately impacting low- and middle-income countries (LMIC), especially those in southern Africa, the Caribbean and parts of Asia, what is known about the effectiveness of western-based interventions in these culturally, racially and economically disparate settings?* With the exciting proliferation of interventions, federal agencies in the USA and international agencies including Joint United Nations Programme on HIV/AIDS realized the importance of assessing the research portfolio and developing metrics of effectiveness. The questions during this phase included: *What is an “effective” intervention? How are effective interventions implemented in a new setting?* This phase merged with the next phase as researchers and public health workers realized that the dissemination to a new community of an intervention developed and found to be effective in one community requires change. The central questions during this time included: *What changes or kinds of changes can be made to an intervention without undermining its effectiveness? What aspects of an intervention cannot be changed without potentially undermining its effectiveness? What constitutes a “change”? Who should be involved in this decision-making?* These efforts culminated in our current phase, one focused on implementation. We must learn more about the factors that allow an intervention to survive and thrive and selectively target these critical factors. The main objective of this paper is to review our experiences and lessons learned in developing, implementing and evaluating FOY in a wide range of socio-cultural settings over the past quarter of century.

## The context in which the Focus on Youth research effort was begun

The 1980s witnessed a world drowning in the wake of the surging Human Immunodefiency Virus (HIV) epidemic. By the late 1980s the causative agent had been identified and modes of infection were understood, but modern medicine had little to offer in terms of prevention or treatment. Biomedical scientists were furiously working to understand this virus and develop drugs and vaccines that could slow or reverse its effects and ultimately prevent its spread (Brandt, 2013).

Until this time there had been relatively little interaction among behavioral scientists, public health scientists and biomedical scientists; the era of multidisciplinary teams was starting, but there was only limited experience with interdisciplinary work. As an early step in this direction, clinical, public health, behavioral and anthropologic communities began working together to develop and implement a range of behavioral interventions designed to impact behaviors to decrease transmission of the disease and decrease its devastating impact.

Care providers and researchers with a particular interest in children and adolescents realized that beside the psychological and cultural aspects to be considered in creating public health interventions targeting children, adolescents and young adults, developmental aspects must also be considered.

In 1988, a group of researchers with diverse disciplinary backgrounds began work in Baltimore, Maryland, on an HIV prevention intervention initially known as Focus on Kids and later renamed as Focus on Youth (FOY). (To avoid confusion, in this report, we shall refer to all of the activities as FOY.)

Over the next 25 years the questions being addressed have changed considerably, informed by the global collective research experience. FOY has had the privilege of evolving through these many phases.

## HIV intervention development: creating the portfolio of prevention interventions

The initial members of the FOY team included anthropologists (with vast experience in participant observation, focus group discussion, semi-structured individual interviews and quantitative ethnographic techniques such as pile-sorting (Stanton, Aronson, Borgatti, Galbraith, & Feigelman, 1993; Stanton, Black, Kaljee, & Ricardo, 1993); psychologists (with an understanding and familiarity with theories of behavioral changes, survey construction and developmental aspects of children and adolescents (Black, Ricardo, & Stanton, 1997; Li, Howard, Stanton, Rachuba, & Cross, 1998; Romer et al., 1994; Stanton et al., 1995); community-based health educators (with an understanding of community involvement in intervention development) (Galbraith et al., 1996); pediatricians (with an understanding of the medical aspects of health and disease in children and adolescents) (Feigelman, Li, & Stanton, 1995; Stanton, Li, Galbraith, Feigelman, & Kaljee, 1996); and statisticians (with the ability to analyze a wide array of longitudinal data and expertise in instrument development) (Stanton, Li, Black, et al., 1996). What each of these professional disciplines of the members of the team lacked was a substantial history in working across disciplines; what each of the members of the FOY team had in common was the realization that we must learn to work together for in isolation, our skills were inadequate to address the epidemic.

During our initial years of work, the questions being addressed by the broader research community included: *Can HIV risk behaviors be purposefully impacted by behavioral interventions? If so, how do successful interventions differ from those that are not successful? How do we define and measure success? Are theory-based interventions more likely to be successful than information-only-based interventions? Can theories be translated into culturally and developmentally appropriate interventions including those that are appropriate for children and adolescents? Do we involve parents and if so, how?* During this phase of FOY, our work was done in collaboration with the communities and their representatives and the residents themselves, both children and parents, living in and around the public housing developments in Baltimore, Maryland, USA. Our target audience was early to mid-adolescents, who are capable of reasoning about hypothetical dilemmas as well as adults but draw on different data banks as they are experimenting with new behaviors with varying potential for risk but do not possess the experience to adequately estimate the consequences of these behaviors. While these age-related characteristics may predispose them to risk, their individual exposure varies as a result of genetics, family and other environmental influences (Cauffman & Steinberg, 2000; Jacobs-Quadrel, Fischoff, & Davis, 1993). Thus, we viewed our task as creating an intervention that was developmentally targeting mid-adolescents but addressed a range of risk exposures prevalent in their communities and propensities based on personal and familial characteristics about which we would have limited knowledge.

**Figure 1.**
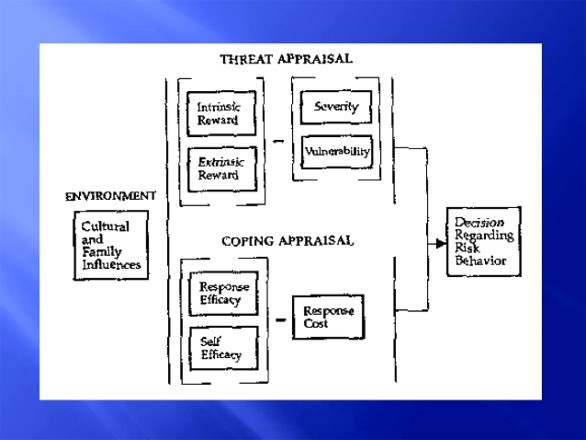
Diagram of PMT. PMT (Rogers 1983) is a social-cognitive model that posits a balance between the *threat appraisal pathway* and the *coping appraisal pathway* in deciding whether to engage in a risky or protective behavior. In assessing the “threat”, we consider the benefits of the risk behaviors: intrinsic rewards (Sex feels good; I want to be intimate with my partner); extrinsic rewards (Having sex make me cool) minus the perceived severity of the consequences of sex (Getting my partner pregnant would not be so bad; getting HIV would be bad) and one's vulnerability (My partner has a reasonable chance of getting pregnant, I have a low chance of acquiring HIV). In assessing the potential “coping” response, we assess the effectiveness of the possible strategy (Condoms are an effective way to prevent HIV if you are going to have sex) and one's ability to perform the protective action (I could buy a condom; I could put a condom on; I am not sure if I could ask partner to use a condom) minus the Response Cost (My friends say condoms take away the pleasure of sex; My partner might think I did not trust her if I asked her to use a condom).

We selected a social cognitive model, Protection Motivation Theory (PMT; Rogers, 1983), appealing for its balance of risk and protective considerations. Because we wanted to emphasize to the youth that actions should be based on decisions, we wanted a model that supported a decision-making framework; PMT lends itself very well to such an approach. Figure 1 provides a graphic illustration of PMT and an example of the application of the theory to a specific decision a hypothetical boy is making about having sex and whether to use a condom.

Guided by PMT, we began our intervention development by two years of participant observations in which we regularly volunteered in the public housing developments and recreation centers in which we would be conducting our research. We maintained field notes and began to construct semi-structured interviews for focus group discussions and individual interviews. The group and individual interviews included various hypothesis generating and testing exercises such as rank-ordering (listing items in order of importance, risk, appeal, etc.) and pile-sorting (arranging items in clusters and then explaining the characteristics in common which resulted in their shared groupings) (Stanton, Aronson et al., 1993; Stanton, Black et al., 1993).

These exercises, followed by an iterative process of pilot testing, enabled us to draft both a risk/protective assessment tool, the Youth Health Risk Behavioral Instrument (YHRBI) and the FOY intervention. The resulting YHRBI consisted of approximately 200 questions each of which was relevant to one or more of the eight constructs of the PMT; therefore, we would be able to address not only whether the intervention worked (e.g. increases in knowledge, protective intentions and behaviors) but also how it was working (e.g. which of the eight constructs had shown improvement – such as increased self-efficacy to perform the protective maneuver or decreased perception of external rewards to partake in the risk behavior) (Stanton, Li, Ricardo, et al., 1996).

**Figure 2.**
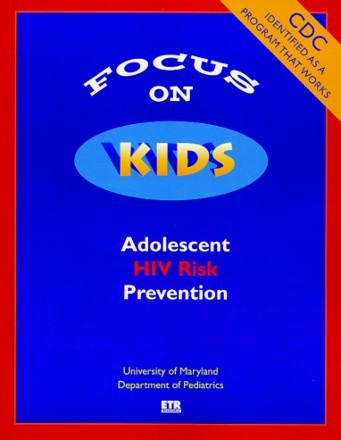
Front cover of “Focus on Kids” curriculum (published by ETR Associates).

The resulting FOY intervention was eight sessions (each session about 75 minutes in length), each session addressing several of the PMT constructs through games, storytelling, demonstrations and factual presentations. Unique aspects of the curriculum including the requirement that the youths develop community projects to practice working with others (Self-perception Theory), delivered to “natural friendship” groups, a story about a fictional family is used to contextualize decision-making and risk that appeared throughout the curriculum, discussion and application of a decision-making model to various situations throughout the curriculum. The front cover of the Focus on Kids training manual is shown in Figure 2.

FOY was assessed through a randomized, controlled trial of 76 naturally formed peer groups consisting of 383 (206 intervention and 177 control) African-American youths 9–15 years of age recruited from six public housing developments in Baltimore, Maryland. The YHRBI which assessed perceptions, intentions and self-reported sexual behaviors was administered to all subjects at baseline (pre-intervention), 6, 12, 18, 24 and 36 months later (Fang, Stanton, Li, Feigelman, & Baldwin, 1998; Li, Stanton, Feigelman, & Galbraith, 2002). In brief, self-reported condom-use rates were significantly higher among intervention than control youths (85% vs. 61%; *p* < .05) at 6-month follow-up, although the difference was no longer significant at 12 months. The intervention impact at 6 months was especially strong among boys and among early teens (13–15 years old). Self-reported condom-use intention was also increased among intervention youths at 6 months but not at 12 months. Following a booster administered at 15 months and again at 27 months, intervention impact again became significant. The conclusions from this trial were that a theory-based and culturally and developmentally appropriate behavioral intervention delivered to small groups of youth can increase adolescent protective behaviors and reduce risk perceptions, intentions and some behaviors for periods up to six months but require additional boosters to be sustained.

Unanticipated were qualitative findings from both the youth and their parents throughout all phases of the study: parents would like to be able to be involved but lack the specific knowledge and skills to conduct conversations with their children on topics addressing sexuality. Accordingly, the FOY team undertook the development of a parental monitoring intervention, using a similar approach to that described earlier for the development of FOY. Ultimately in collaboration with a professional videographer (Video Press, Baltimore, Maryland), the resultant intervention (Informed Parents and Children Together (ImPACT)) included a 22-minute video (*Protect Your Child from Acquired Immunodeficinecy Syndrome (AIDS)*) followed by a condom demonstration involving the parents and two role plays with six simple messages: (1) talk to your children about sex before they have sex; (2) talking about sex is awkward – at first; (3) know where your child is, with whom your child is with and what your child is doing; (4) condom-use skills; (5) communication skills are important and require listening and practice; and (6) your child wants to talk to you (Li, Feigelman, & Stanton, 2000; Romer et al., 1999; Yang et al., 2006).

The effectiveness on ImPACT on both parent and youth outcomes was assessed two and six months post-intervention through a randomized controlled trial of 237 parent–youth dyads who were randomized at the level of the dyad to receive ImPACT or a dose-matched control condition, a goal setting video developed for parents and youth. (The control video was developed after conversations with community members as to other skills that they would find useful.) At baseline, parents significantly underestimated youth's risk behaviors; but two and six months post-intervention, parents and youth receiving ImPACT compared to controls demonstrated significantly increased similarity of youth involvement in risk and protective behaviors. Through six months post-intervention, ImPACT youths and parents demonstrated higher levels of condom-use skills, but no significant impact on youth risk behavior was found, leading us to speculate that a parental monitoring intervention such as ImPACT should be given to parents in conjunction with more traditional youth-centered risk-reduction interventions (Stanton et al., 2000).

Accordingly, we undertook a randomized, controlled trial involving 817 African-American mid-adolescents from 35 low-income sites in and around Baltimore, Maryland. Because FOY had been demonstrated to reduce risk behaviors through six months post-intervention, all youth participated in the eight-session, theory-based, small group, risk-reduction intervention. In addition, 496 youth and parents received the one-session ImPACT intervention, while the remaining 321 dyads received the one-session Goal for It attention-control condition. The YHRBI was administered to the youth to assess risk and protective behaviors, perceptions and knowledge at baseline, 6, 12 and 24 months. At six months post-intervention, FOY plus ImPACT reported significantly lower rates of sex, sex without a condom, alcohol use and cigarette use compared to FOY plus Goal for It. *At 24 months post-intervention*, 6 of 16 risk behaviors were significantly reduced among FOY plus ImPACT youth compared to 4 of the 7 theory-based subscales reflecting significant protective changes (Li, Stanton, & Feigelman, 2000; Li, Stanton, Galbraith, et al., 2002; Stanton et al., 2004; Wu et al., 2003).

Importantly for the national efforts to reduce HIV transmission, several federal and other national groups had developed processes to identify behavioral interventions with credible evidence of effectiveness in reducing risk behaviors and/protective factors associated with risk behaviors. Particularly notable among these efforts was that of the Centers for Disease Control and Prevention (CDC). Specific to adolescent risk reduction, the CDC's Division of Adolescent Health identified through several levels of analysis conducted by CDC staff and external panels of experts six “Programs that work”, evidence-based HIV risk-reduction interventions targeting adolescents (Collins et al., 2002). These programs were then packaged for dissemination in partnership with professional health educator national agencies. FOY was included in both of these portfolios and in collaboration with Educational Training Resources (ETR) Associates (Scotts Valley, California: Pub.etr.org) two national trainings (reaching 64 trainers from across the USA) were conducted. FOY was identified in several other US sites identifying effective programs including SAMHSA's National Registry of Effective Programs, the Urban Institute's “Teen Risk Taking: Promising Prevention Programs and Approaches” and the US Office of Population Affairs' Program's “Archives on Sexuality, Health and Adolescence”. By the later 1990s, to our knowledge there were sites in 12 states throughout the USA in which FOY had been offered to at risk youth (Galbraith, 2004; Galbraith et al., 2009). The FOY team itself conducted a longitudinal trial of a rural adaptation of FOY in West Virginia (D'Alessandri et al., 2003; Stanton et al., 2005, 2006). While some of the groups and individuals adapting FOY to new settings conducted randomized controlled trials to evaluate the program and published the results (e.g. Bell et al., 2007; Gaydos et al., 2008; Morrison et al., 2007), although we suspect that the majority did not conduct effectiveness trials.

The “big picture” findings from this phase of research included the following: (1) anthropologic and psychological approaches can be employed to produce an effective theory-based, culturally and developmentally based intervention; and (2) youth and parents desire parental involvement in sexual risk-reduction efforts and their involvement augments the impact of the intervention.

## The global epidemic: adapting effective interventions to new settings

Although gratified with our US-based efforts and results, the FOY team had increasingly become troubled by a disturbing reality. A large number of interventions had been developed and some had been shown to have evidence of impact. But virtually all of these interventions – and especially those that had been evaluated – had been conducted in the USA or Europe. By the end of the twentieth century, 94% of persons infected with HIV were living in the developing world; 86% of those infected lived in sub-Saharan Africa, home to only 9% of the world's population. In several of these countries, the sero-prevalence exceeded 25% (Iliffe, 2006). And, closer to home of the USA, two countries in the Caribbean had rates in excess of 4% and 5% (Wheeler & Radcliffe, 1994). Particularly concerning the FOY group, youth between the ages of 10 and 24 accounted for more than 50% of new post-infancy infections worldwide.

The questions facing researchers during this phase included: *With the global burden of HIV disproportionately impacting low- and middle-income countries (LMIC), especially those in southern Africa, the Caribbean and parts of Asia, what do we know about the effectiveness of western-based interventions in these culturally, racially and economically disparate settings?* Many of us were being asked to bring our interventions to these different settings. *What is the process for adapting effective interventions to new settings? How do researchers from different continents form partnerships with communities without living and working together?* During this phase, we worked (and continue to work) with many communities across the globe in Africa (Namibia), Asia (China and Vietnam) and the Caribbean (The Bahamas, Tobago and Trinidad).

The issues confronted and subsequent journeys of the FOY curriculum and team in these settings varied tremendously. In two settings (Namibia and The Bahamas), the thrust of our efforts remained directed toward adolescents; accordingly, these are the international efforts which will be featured in this manuscript. In China, although several trials were conducted among adolescents (Cottrell et al., 2005; Li et al., 2004, 2008; Zhang, Li, Shah, Baldwin, & Stanton, 2007; Zhang et al., 2004, 2010) given the age of sexual initiation, ultimately we felt our efforts were more appropriately directed toward young adults. These efforts (with examples of relevant publications) include rural to urban migrants and sex workers (Hong et al., 2006; Li et al., 2006; Liu et al., 2006; Yang et al., 2004) and other issues of great importance requiring research attention such as children whose parents were living with or had succumbed to AIDS (Hong et al., 2011; Lv et al., 2010; Tu et al., 2009; Yu et al., 2013; Zhao et al., 2007, 2009). Likewise in Vietnam, with an older age of sexual initiation, our efforts were focused on young adults (Kaljee et al., 2005, 2011). Therefore, since this manuscript is focused on adolescents, we shall not describe the efforts in detail here.

Although the age ranges were similar to those that we had addressed in the USA, in Namibia and The Bahamas there were many aspects of the situations which were quite different. Therefore, in both settings we elected to conduct ethnographic and some survey work to adapt the curriculum to these different settings.

## Namibia

Our first experience was in Namibia. Namibia achieved independence from South Africa after a protracted war ending in 1990. In 1993 the First National HIV Prevalence Study was conducted and found the sero-prevalence to be 5%. The results were met with shock and uncertainty and so the survey was repeated in 1994, revealing a sero-prevalence of 8%. The third such study was conducted in 1996, revealing an overall rate of 15%, with the rate as high as 25% in Caprivi (Fitzgerald et al., 1999). The United Nations Children's Fund (UNICEF) Mission Director was aware of FOY and invited us to work with the Ministry of Health, of Youth and Sport and UNICEF to develop FOY to an intervention appropriate for Namibia.

The Ministry of Education and UNICEF made the decision to focus the initial efforts in two districts with especially high rates of HIV. Efforts to determine if FOY were appropriate for the Namibian setting including conducting focus group discussions and some individual interviews among students in the upper school (high school). The ages of these students varied considerably with most of the females being in their mid- to late teens, while the males also included former freedom fighters in their 20s who had returned to complete their education after independence. Using the information obtained from these interviews, we adapted the YHRBI to better reflect the issues facing these youth in Namibia, but maintained the general structure of the questionnaire, including the underlying theory (PMT) which appeared to be consistent with their decision-making frameworks. The revised instrument (renamed by the Namibian partners of the team to “Towards a Healthy Namibia”) was administered to 922 youth. The results generally confirmed the applicability of PMT and of the general FOY curriculum, but identified many differences which required adaptation of the curriculum including significant differences in perceptions and risk behaviors by gender, very limited knowledge regarding HIV, limited access to condoms throughout the nation and very high use of alcohol (Stanton et al., 1999). The resulting Namibian version of the intervention was renamed “My Future is MY Choice!” including six additional sessions to accommodate new facts, more local stories to illustrate points, and sessions regarding gender inequality and alcohol use. The same format of stories, discussions, vignettes and time for questions was retained with an emphasis on decision-making, condom skills, negotiating skills and gender rights (Fitzgerald et al., 1999; Stanton et al., 1998). A randomized, controlled trial was conducted among 515 (262 intervention) youth in 10 secondary schools. Although it was delivered after school, there was an 80% participation rate among the students. As we had found in the prior survey, there were highly significant gender differences across the majority of variables. There were significant increases in knowledge skills, perceptions and intentions. Use of condoms remained very low at least in part due to lack of access (Fitzgerald et al., 1999; Stanton et al., 1999). The Ministry of Health and UNICEF devised a strategy to increase the supply of condoms throughout the nation based on these results. The subsequent story of “My Future Is MY Choice” is very heartening. The curriculum is owned by Namibia. Last posting by UNICEF (1999, 2007), the curriculum was implemented in middle and high schools through Namibia to over 160,000 students. It was identified by Joint United Nations Programme on HIV/AIDS as a “Best Practices” HIV curriculum in 2000. In a subsequent “south-to-south” transfer, Namibian educators taught the intervention to Mozambique educators who implemented it in 13 districts through the Ministry of Youth and Sports. The last posting on the internet indicates that 15,000 Mozambique youth had been trained in the intervention (UNICEF Mozambique, 2004).

### The Bahamas

Halfway around the world from Africa, the Caribbean was also struggling with the HIV epidemic. In the mid-1990s, the sero-prevalence in the recently independent Commonwealth of The Bahamas approached 5%. The government aggressively mounted an impressive effort to combat the epidemic (Wheeler & Radcliffe, 1994). In 1998, the government learned about FOY and invited members of the team to work with the Ministry of Health to adapt FOY for use in The Bahamas. The adaptation, resulting in a 10-session intervention with the 2 additional sessions addressing alcohol use, was initially pilot-tested among adolescent females attending a school for pregnant teens.

The pilot test indicated that the program was effective, but the Ministries of Education and Health jointly decided that a more effective national strategy would be the development of a program targeting younger youth, ideally before they had initiated sex. The program was further modified to be developmentally appropriate and was named Caribbean Focus on Youth (FOYC). While FOYC was well-received in focus groups and a pilot test among grade 6 students, the students, parents and teachers indicated a preference to have a companion parent intervention (Yu et al., 2006). They were interested in a program similar to the US-based parental monitoring intervention “ImPACT” described earlier, but wanted an approach which better reflected the developmental needs of the younger age of the youth (preteens rather than teens) and the Bahamian culture. Similar to ImPACT, the new parent intervention, *Caribbean Informed Parents and Children Together* (CImPACT) was built around a video (*Keeping the Promise*) lasting about 25 minutes. (Click link to view segments of *Keeping the Promise*, https://www.dropbox.com/s/gq5v6o2079y2f0t/VTS_01_1.VOB.) This video, filmed in The Bahamas, was adapted to be culturally appropriate for The Bahamas and developmentally appropriate for the age of the target children (pre-adolescence, rather than early–mid-adolescence). Although the general messages regarding parenting were similar to the original US-based version, they were more appropriate to the younger youth and reflected the local culture. While ImPACT was delivered to the parent–youth dyad, CImPACT was delivered to small groups of parent–youth dyads. Similar to ImPACT, a condom demonstration and role play followed the showing of the video; in addition, a 15–20-minute discussion followed. The Bahamian government wished to conduct an effectiveness trial of FOYC and CImPACT. The same parent control that had been used in the USA (the job skills preparation entitled “Goal for it”) was felt by participants in focus groups to be suitable for use without remaking it. However, for the control comparison for FOYC, The Bahamian Ministry of Education desired a program that itself would be of benefit to the youth and requested an environmental program. In collaboration with an ecology consultant, the West Indian Whistling Duck and Wetlands Conservation Project and the Bahamian Land Trust, we designed a dose-equivalent environmental conservation intervention targeting Bahamian waterways and marshes entitled the Wondrous Wetlands.

**Figure 3.**
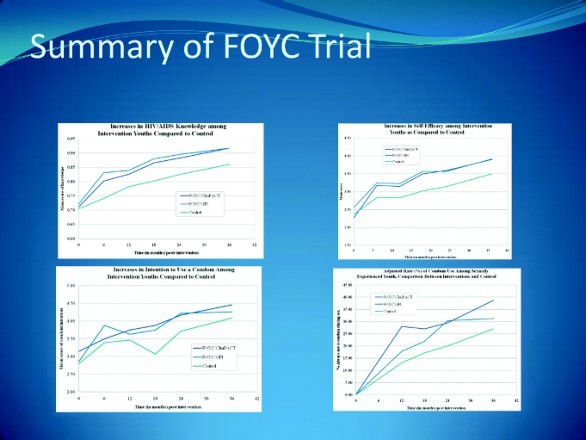
Impact of FOYC in four domains over 36 months.

In collaboration with The Bahamian Ministries of Health and of Education, the FOY team conducted a three-celled randomized controlled trial among 15 of New Providence's 26 government elementary schools involving 1360 students and their parents of the estimated 1900 students attending the schools. Five schools each were randomized to receive *FOYC plus CImPACT* (436 youth and a parent), *FOYC plus Goal for It* (427 youth and a parent) and the *Wondrous Wetlands plus Goal for It* (497 youth and a parent). Six assessments were performed (baseline through 36 months follow-up) employing a Bahamian version of the YHRBI. As shown in Figure 3, youth receiving FOYC (and especially the youth randomized to receive CImPACT) demonstrated consistently higher knowledge, intentions to use a condom if they were to have sex, their self-efficacy regarding condom use and at 36 months post-intervention, higher condom-use rates (Chen et al., 2009, 2010, 2012; Deveaux et al., 2007). As in our previous work, besides assessing intervention impact, the data collected permitted us to explore many related aspects of sexual decision-making including peer, neighborhood and parental influences (Liu et al., 2007; Stanton, Jones, et al., 2012; Wang et al., 2013; Yu et al., 2008), the development of assessment instruments (Deveaux et al., 2007; Stanton et al., 2009) and the exploration of the many experiences gained through complex, community- and school-based research (Deveaux et al., 2011). We are currently completing a randomized controlled trial examining the impact of a modified version of CImPACT administered to grade 10 students. Utilizing all eight government high schools on the island of New Providence, we are able to examine the impact of the modified version of FOYC administered at this grade level (with or without CImPACT) among naïve grade 10 students (e.g. those who had no prior exposure to FOYC) but also the impact on those who previously had received FOYC in grade 6. Because several hundred of the students were enrolled in both the grades 6 and 10 studies, we were able to determine that the intervention was still impactful four years later (Stanton, Chen, et al., 2012). Through a series of analysis examining various combinations of the grade 10 intervention status and that of the grade 6 study, we were able to determine that six months post-Bahamian Focus On Older Youth (BFOOY) intervention, receipt of BFOOY by youth who had *not* received FOYC in grade 6, resulted in increased HIV knowledge and condom-use skills. By contrast BFOOY grade 10 youth who *had* received FOYC in grade 6 already had increased knowledge and condom-use skills at baseline compared to those of the unexposed grade 10 youth; these youth did not receive a significant increase from the additional exposure to BFOOY. However, if instead of controlling for baseline differences between the two groups in grade 10 (since the differences did result from prior exposure among one group to the intervention), the youth receiving both interventions (e.g. FOYC in grade 6 and BFOOY in grade 10) displayed both a carryover effect from the FOYC and an additional boost from BFOOY, and thus demonstrated the highest scores six months post-intervention in condom-use skills (Dinaj-Koci et al., 2012). These data provide support for the importance of continued teaching of the curriculum in grade 6, but also suggests that the second exposure in grade 10 to the curriculum serves a useful and valuable purpose: it has a significant impact on youth who missed the grade 6 exposure and serves to effectively boost the skills of the youth who did receive FOYC back in grade 6.

#### What happens after we leave?

This work was remarkably exciting and gratifying. It was very fulfilling to know that at some time our efforts had touched children, adolescents and parents in settings across the globe. Still, many times we wondered – what happened after we left? Were our former research trainers still using the curriculum? One member of our research team was a health educational doctoral student at the time who elected to conduct her dissertation exploring sustained implementation and fidelity of the multiple FOY trainings around the globe. She identified over 300 individuals who had been trained to deliver FOY in some venue in multiple sites across the globe; through a decision-making tree she sought to interview the 43 trainers she identified who had implemented FOY within the past year. She was able to administer a cross-sectional telephone survey to 34 (73%) of the targeted individuals, assessing how the interviewer or organization had adhered to, adapted, dropped or altered the intervention. None of those interviewed had implemented all of the critical elements of the intervention (“Core Elements, *vide infra*). She concluded that more effort is needed to reflect the constraints practitioners face in non-research settings (Galbraith, 2004; Galbraith et al., 2009). Her work, conducted about a decade again, helped to set the stage for the next phase of intervention focus at a national and global level, identifying and preserving the components of effective interventions.

## Bringing evidence-based interventions to the community

As more and more HIV prevention interventions were developed through the decade of the 1990s, public health professionals and researchers contemplated the next steps for the prevention field. While countless HIV prevention programs had been developed, many had never been evaluated, and some that had been evaluated had not been shown to be effective but were still being implemented and/or disseminated. With the exciting proliferation of interventions, federal agencies in the USA and international agencies realized the importance of assessing the research portfolio and developing metrics of effectiveness. At a broad level, the goals were to enable communities and agencies desiring to protect their residents, members or students to be able to select an intervention that had already been developed and found to be effective and to guide them as to which effective intervention might best suit their needs. The questions during this phase included: *How do we – and who is “we” – determine what an effective intervention is? How – and who – trains new communities in the implementation of an effective intervention in a new setting?* As part of this response, the CDC created the Prevention Research Synthesis program whose charge was to convert evidence from the published research literature into practical information accessible to prevention providers, researchers and health departments (local and state) throughout the USA and beyond. CDC staff and panels of experts conducted rigorous, structured efficacy assessments in which they adhered to predefined strict criteria defining criteria for the evidence base; only those programs achieving one of two levels of evidence (*Best* and *Good Evidence*) were accepted. First published in 1999 with periodic updates thereafter, the evidence-based interventions (EBIs) were listed and described in the *Compendium of HIV Prevention Interventions with Evidence of Effectiveness. The Risk Reduction Chapter of the Compendium* included valuations of the interventions, had to have been published in a peer-reviewed journal, been identified through systematic search, focused on an HIV, AIDS and/or Sexually Transmitted Disease (STD) behavioral prevention intervention, included a comparison condition, been conducted in the USA and report *at* least one behavior directly impacting HIV or biologic measures of HIV or other STD infections. At its most recent update in 2011, the Risk Reduction chapter included 74 EBIs: 44 *Best*-Evidence and 30 *Good*-Evidence interventions. FOY plus ImPACT was selected as one of the Best-Evidence EBIs. (See http://www.effectiveinterventions.org for more details.)

What this Compendium does offer as a stand-alone product is an easily accessible list of interventions determined to be effective along clearly defined criteria and contained a brief description of the target audience, format and content of the intervention. What it does *not* accomplish is the provision of a manual useful for interested groups to guide and facilitate their implementation efforts. Therefore, despite a directory of the many well-evaluated interventions found to be effective, there was still limited utilization of EBIs in the field. (For more information, see www.cdc.gov/hiv/dhap/prb/prs/index.html.)

Accordingly, the Capacity Building Branch, Division of HIV/AIDS, Center for Diseases and Prevention instituted a companion program, the Diffusion of Effective Behavioral Interventions (DEBI) program (Collins, Harshbarger, Sawyer, & Hamdallah, 2006; Dolcini et al., 2010; Lyles, Crepaz, Herbst, & Kay, 2006; McKleroy et al., 2006). The DEBI program was designed to define and implement a national-level plan for the training, ongoing field support and general trouble-shooting to support the implementation of evidence-based HIV prevention interventions to state and community HIV programs throughout the USA. To be included as a DEBI program, an intervention had to be a Best-Evidence EBI, the CDC staff needed to determine that the intervention could be packaged, and the developers needed to be willing to have the program packaged and to participate in the effort. FOY with ImPACT was selected into the DEBI program. (For more details, see www.effectiveinterventions.org/en/home.aspx.)

ETR Associates was selected to serve as the implementing partner for the DEBI roll-out. (As noted above, ETR had also been our partner for the roll out of FOY as one of the *Programs that Work* discussed earlier.) The packaging of the program included refilming the ImPACT video as it was felt to be somewhat local in nature and the creation of program worksheets for the students to facilitate intervention implementation for the trainers. Prepared by professional trainers and approved by researchers, a specific curriculum manual was produced to teach the trainers how to implement the curriculum. Training sessions (“institutes”) were scheduled to provide direct training to future trainers of trainers and regional, state and local trainings were provided to diffuse the intervention throughout the USA. Researcher involvement was encouraged (and we did participate) in the train-the-trainer sessions which included intensive skill-building around the intervention, the establishment relationships between the trainers and his/her coach and specific implementation plans. A variety of trainer support mechanisms were offered to the trainers for use after the training session was over including online training (primary or secondary), satellite broadcasts, list serves, newsletters, follow-up calls, etc. This experience underscored the importance of an active partnership with excellent communication at all stages among researchers, agencies concerned with public health (such as the CDC), the implementing partner (such as ETR) and the actual implementers (the trainers). Implementation of an effective intervention requires multidisciplinary effort among community change agents, curriculum writers, trainers, those providing backup to the trainers, the researchers and agency administrators (Dolcini et al., 2010; Stanton et al., 2007).

## Intervention implementation research: the new frontier

These efforts culminated in our current exploration, an implementation phase. HIV continues to impact LMIC nations as well as financially and socially stressed populations within industrialized nations. There is no question that behavioral interventions – and several very powerful biomedical interventions – can dramatically reduce the transmission of HIV and the impact of HIV infection on an individual. However, we and others interested in implementing effective interventions have come to realize several other realities. First, adaptation of interventions as they are implemented by different trainers and/or in different sites is inevitable. Adaptation is also necessary as we learned each time we sought to implement FOY in a new setting. What we do not know is: *How much adaptation is too much? Are there “Core Elements” or components of an intervention that cannot be eliminated or altered?* (Stanton et al., 2011)

It is equally clear that the resources invested to maintain interventions at an effective level using external reinforcements are beyond the financial resources of most communities and certainly LMIC communities. Therefore, we must learn more about the factors that allow an intervention to survive and thrive and selectively target these critical factors. This is the heart of implementation research and represents our current research focus, whose research platform is in The Bahamas with their national implementation of FOYC throughout all government grade 6 Bahamian classrooms.

Our current effort is based on the principles of implementation *science* and seeks to further develop this field of science. Implementation science is primarily concerned with the process of delivery and how this process impacts anticipated outcomes based on prior effectiveness research (Dearing, 2009). In our ongoing research in the Bahamas, the Ministries of Education and of Health are hopeful that the nationally implemented FOYC plus CImPACT program will remain as effective in increasing student knowledge and protective skills, increasing protective perceptions and intentions and increasing condom use as it was during the effectiveness trial (Chen et al., 2010; Stanton, Chen, et al., 2012). National implementation of an evidence-based behavioral intervention offers a unique opportunity to contribute to the emerging field of behavioral implementation science (McKleroy et al., 2006; Rogers, 2010). *Fidelity* of behavioral implementation refers to the degree to which program implementers (“trainers” who in The Bahamas are the grade 6 teachers) actually do implement the program (e.g. do teach FOYC) and implement it as intended by the program developer (Dearing, 2009); in the present case, the developers of FOYC intended that all 10 sessions of the FOYC curriculum be taught, including all components of activities in each session. However, a robust literature regarding implementation of education and behavioral interventions confirms what we have stated earlier in this section: some degree of alteration is unavoidable (Bellg et al., 2004; Dusenbury, Brannigan, Falco, & Lake, 2004; Ringwalt, Vincus, Ennett, Johnson, & Rohrbach, 2004; Rohrbach, Ringwalt, Ennett, & Vincus, 2005). At the same time, there is a small but compelling literature that the closer intervention delivery is to that shown to be effective, the closer the outcomes will be. A particularly comprehensive examination of fidelity and effectiveness assessing seven nationally disseminated education and criminal justice projects found that implementations conducted with high-fidelity (few changes) were more effective than low-fidelity (many changes) implementations (Blakely et al., 1987). They also observed that local additions to the model appeared to increase effectiveness or were unrelated to effectiveness.

To enable trainers of a curriculum in a new setting to make some adaptations without impairing the effectiveness of the intervention, the DEBI program has worked with researchers to explicitly identify what they believe are the *Core Elements* or critical characteristics of the original (effective) intervention without which it is questionable whether the intervention would retain its effectiveness (McKleroy et al., 2006). Core Elements can be considered in two categories: *Implementation-Pedagogy* (characteristics relating to the logistics of establishing a positive learning environment and the teaching process used); and *Content* (the information, skills, values, etc. that are being taught). Each of these components is considered critical in diffusion/adaptation of interventions. At the time that the CDC had accepted FOY with ImPACT into the DEBI program, we were working with researchers in The Bahamas to adapt the intervention for grade 6 students in the largest of the Bahamian island, New Providence. To identify the Core Elements of FOY with ImPACT, we used the three-step process suggested by Kelly et al. (2000): (1) examine the behavioral science theory; (2) use the experience and feedback from participants and experienced program staff about what activities were most effective and (3) derive insights from controlled experiments of the intervention. Identifying the Core Elements of FOY with ImPACT involved the developers, the CDC and the DEBI implementers and occurred over many months. (See Table 1 for Core Elements of FOY with ImPACT.) Therefore, in the adaptation of FOY with ImPACT to the Bahamian version (FOYC plus CImPACT), the US-Bahamian researchers were careful to adhere to as many of the identified Core Elements of both FOY and ImPACT as considered appropriate in the Bahamian community. Therefore, the Core Elements for the adapted intervention used in The Bahamas remain very similar to those identified for the original DEBI program.

**Table 1.** FOY with ImPACT Core Elements.

Implementation Core Elements: FOY
*Core Element 1*: Deliver intervention to youth in community-based settings
*Core Element 2*: Use two skilled facilitators to model communication, negotiation and refusal skills for the youth
*Core Element 3*: Use “friendship” or venue-based groups (i.e. a basketball team, a scout troop, church group, an existing youth group) to strengthen peer support
Content Core Elements: FOY
*Core Element 4*: Use culturally appropriate interactive activities proven as effective learning strategies to help youth capture the important constructs in the theory
*Core Element 5*: Include a “family tree” to contextualize and personalize abstract concepts, such as decision-making and risk assessment
*Core Element 6:* Enable participants to learn and practice a decision-making model such as SODA (Stop, Options, Decide, Action)
*Core Element 7*: Train participants in assertive communication and refusal skills specifically related to negotiation of abstinence or safer sex behaviors
*Core Element 8*: Teach youth proper condom-use skills
Implementation Core Elements: ImPACT
*Core Element 1*: Delivering intervention *one-on-one* to parents/guardians and youth in community-based setting or home at a time and place that is convenient for parent/guardian
*Core Element 2*: Use of a facilitator whom the parents/guardians find credible. Facilitator should be skilled at building rapport with parent and youth at the beginning of the session
*Core Element 3*: *ImPACT* should be delivered prior to or soon after the youth begin the *FOY* intervention
Pedagogy Core Elements
*Core Element 4*: Use the Protect Your Child from AIDS video that shows the challenges and importance of parents talking to their children about sex, abstinence, STDs, etc.
*Core Element 5*: Facilitator must sit down and watch the video with the parent/guardian and youth. Youth and parent/guardian must watch the video together
Content Core Elements
*Core Element 6*: Enabling parent and youth to learn and practice communication skills
*Core Element 7*: Teaching parent/guardian and youth proper condom-use skills
*Core Element 8*: Distributing and guiding parent/guardian and youth through a Resource Guide that includes the following topics:
• Basic components of good communication and how to talk to your youth
• Importance of parental monitoring
• Steps for proper condom use
• STD and HIV facts, including prevalence data among young African-Americans
Video/DVD Key Messages
(1) *It is important to talk to your youth about sex before they start having sex*
• Best time to influence is before youth start having sex
• Find a good time for you (parent/guardian) and youth
• You cannot wait for them to ask about sex
• Do not wait until he/she is in the situation, because you will not be there!
• Parents need to talk with their youth about STDs and pregnancy
(2) *Parents should talk to their children about abstinence*
• Talking to youth about abstinence and making sure to correct the misperception that “everybody's doing it” will allow them to make better sexual decisions
(3) *It is important to know whom your youth is with, what he/she is doing and where he/she is*
(4) *It is important for youth to know how they would respond* if they were in a situation in which they might be pressured into having sex
(5) *There are serious consequences to risky sexual behavior*
• Although treatment is now available that allows people to live much longer with HIV, there are still many difficulties with being HIV infected
• Sex can make it difficult for a young person to reach their goals
• The decisions youth make when young have an impact on their future
(6) *Parents should talk to their youth about proper condom use, making sure they know how to use condoms*
(7) *Communication goes both ways*
• Be approachable. A parent's negative reaction to a youth coming to talk can stop future conversations
• It is important to listen to your youth
• Often youth wants parents to talk to them about sex. It shows them you care
(8) *Talking with your youth is difficult but it gets easier over time*
• Both parents and youth often feel awkward about these discussions
• Be prepared. Do the best you can do as a parent and for yourself
• It is OK to tell your youth you do not know the answer to a question and to find out the answer later
(9) Parents and youth are having these difficult conversations successfully
(10) *If you feel you cannot talk to your youth about sex, it is important to find someone else to talk to him/her*. Find someone who shares your values and has a good rapport with your youth so the youth respects and enjoys talking with this person
(11) Allow youth to grow toward independence, but set guidelines too
• Ultimately, youth are going to make their own decisions, but it is parents' job to give them information and prepare them as much as possible

To advance our understanding of implementation science and to aid the Ministry of Education of The Bahamas in their national implementation effort of FOYC, the FOY team and our Bahamian researcher partners are conducting (1) an evaluation of the fidelity of the implementation of FOYC plus CImPACT; (2) an assessment of factors potentially influencing fidelity of implementation; (3) a study of the impact of variations in fidelity of implementation on intervention outcomes and (4) exploring issues in the measurement of fidelity. Fidelity and factors potentially influencing fidelity are being assessed through seven measures of implementation (structured observations of the teachers by trained observers and teacher self-reports). The Ministry is administering a curriculum assessment to the students at the beginning and end of grade 6 (pre- and post-curriculum delivery) and again at the end of grades 7–9 which assess knowledge, condom-use skills, perceptions, intentions and some self-reported behaviors. The assessment is totally anonymous (no name or student identification number) as the interest is in curriculum effectiveness, not individual student behavior. These data are being used to assess whether the curriculum remains as effective and to correlate student impact with fidelity of implementation.

The project is being implemented over two waves. The first wave of national implementation (2011) involved 35 government primary schools and 110 teachers. The school received assessments from approximately 2500 grade 6 students both prior to and after the teaching of FOYC (Knowles, Kaljee, et al., 2012; Knowles, Wang, et al., 2012). A summary of our findings reveals that teachers taught a mean of 16.3 out of 30 core activities, 24.9 out of 46 total activities and 4.4 out of 8 sessions. (The Ministry of Education had rearranged the 10-session FOYC curriculum described earlier into 8 sessions; however, the content was not changed.) The strongest predictor of implementation fidelity was teacher comfort with the FOYC curriculum. Teachers who did not perceive the FOYC intervention to be important for their students or who had attended only part of an FOYC training workshop were more likely to change the curriculum. Increased duration of experience as a teacher (>10 years) was negatively associated with fidelity of implementation. Teacher's perception of the importance of the FOYC intervention and implementation fidelity had direct positive effects on students' HIV/AIDS knowledge, reproductive health skills, protective intentions and self-efficacy. Youth did not appear to benefit from FOYC if two or fewer sessions were delivered.

This is an exciting phase for the FOY team as all of the efforts that we and other teams have invested in intervention development and evaluation is of diminished value if our interventions are not adapted, sustained and implemented in a manner that retains initial effectiveness.

### Ruminations

How does one summarize over two decades of work in a single manuscript? The answer is simple; one does not; one only scratches the surface. Still, so many lessons have been learned which are generalizable.

First, parents wherever we have been recognize that they have difficulty talking with their children about sex – but desire the skills to be able to do so. And, in each community we have visited, the youth have been pleased to have these conversations. Parents would endure substantial inconveniences to be able to take advantage of the opportunity to gain these skills and/or to have a guided discussion about these topics with their children.

Second, each community has very different needs, needs which may be difficult to imagine without asking; but regardless of their needs, youth everywhere have questions about sex and diseases related to sex which they want to discuss. Despite a very high HIV sero-prevalence, many youth in Namibia had never heard of HIV. By contrast, despite their very young age, most grade 6 students in The Bahamas were well aware of HIV. Even though youth in Vietnam and China did not engage in sex at an early age, they were very interested in learning about HIV.

Third, communities desire ownership of the curriculum that they develop; they take pride in what they have produced. This is of course a good thing and the more involvement of the community in the adaptation of the curriculum, the more likely it is that the community will regard it as “theirs”. Our failure to substantially involve the community in the development of the West Virginia version of FOY may in part explain why the intervention was less impactful in that setting.

Fourth, at some point, if the process has gone well, the researcher may become accepted by a community. In our experience, this honor is unlikely to be accompanied by formal declarations and therefore the investigator may not realize his or her change in status. It is important to be sensitive to this acceptance for it is accompanied with substantial privilege and considerable responsibility. It is not that we actively or consciously sought acceptance. We did not ask to be accepted; through our actions and interactions, it happened (to one degree or another) in some settings, but not in others. In retrospect, our work was very much less effective in those communities in which we were not accepted. But once acceptance has occurred, the researcher must both be aware (and appreciative) of the honor and be mindful of the responsibilities that accompany such acceptance.

## Conclusions

During each of these phases we have learned much about risk and protective factors at the level of the child, family and community; changes in the constellation of these factors across the adolescent years; and cultural variations in these factors. We have gained increased understanding of the roles of parents and how to enhance their protective effects as well as the role of peers and how to modify their effects. We have learned about the sequencing of interventions across the adolescent years. These findings have real implications and we like to believe that we have made a difference.

But what we know is that FOY – and the individuals with whom we have had the joy of collaborating across the globe – has profoundly impacted us. Behind every description of the various phases of the FOY quarter-century history are countless memories and stories – and a wellspring of gratitude to the hundreds of communities who opened their doors and gave us the gift of partnership.
